# Prognostic value of multi-PLD ASL radiomics in acute ischemic stroke

**DOI:** 10.3389/fneur.2024.1544578

**Published:** 2025-01-13

**Authors:** Zhenyu Wang, Yuan Shen, Xianxian Zhang, Qingqing Li, Congsong Dong, Shu Wang, Haihua Sun, Mingzhu Chen, Xiaolu Xu, Pinglei Pan, Zhenyu Dai, Fei Chen

**Affiliations:** ^1^Department of Radiology, Affiliated Hospital 6 of Nantong University, Medical School of Nantong University, Nantong, Jiangsu, China; ^2^Department of Neurology, Affiliated Hospital 6 of Nantong University, Yancheng Third People’s Hospital, Yancheng, Jiangsu, China; ^3^Department of Radiology, Suzhou Wuzhong People’s Hospital, Suzhou, Jiangsu, China; ^4^Department of Radiology, Affiliated Hospital 6 of Nantong University, Yancheng Third People’s Hospital, Yancheng, Jiangsu, China; ^5^Department of Central Laboratory, Affiliated Hospital 6 of Nantong University, Yancheng Third People’s Hospital, Yancheng, Jiangsu, China; ^6^Medical Imaging Institute of Jiangsu Medical College, Yancheng, Jiangsu, China

**Keywords:** acute ischemic stroke, radiomics, arterial spin labeling, cerebral blood flow, machine learning

## Abstract

**Introduction:**

Early prognosis prediction of acute ischemic stroke (AIS) can support clinicians in choosing personalized treatment plans. The aim of this study is to develop a machine learning (ML) model that uses multiple post-labeling delay times (multi-PLD) arterial spin labeling (ASL) radiomics features to achieve early and precise prediction of AIS prognosis.

**Methods:**

This study enrolled 102 AIS patients admitted between December 2020 and September 2024. Clinical data, such as age and baseline National Institutes of Health Stroke Scale (NIHSS) score, were collected. Radiomics features were extracted from cerebral blood flow (CBF) images acquired through multi-PLD ASL. Features were selected using least absolute shrinkage and selection operator regression, and three models were developed: a clinical model, a CBF radiomics model, and a combined model, employing eight ML algorithms. Model performance was assessed using receiver operating characteristic curves and decision curve analysis (DCA). Shapley Additive exPlanations was applied to interpret feature contributions.

**Results:**

The combined model of extreme gradient boosting demonstrated superior predictive performance, achieving an area under the curve (AUC) of 0.876. Statistical analysis using the DeLong test revealed its significant outperformance compared to both the clinical model (AUC = 0.658, *p* < 0.001) and the CBF radiomics model (AUC = 0.755, *p* = 0.002). The robustness of all models was confirmed through permutation testing. Furthermore, DCA underscored the clinical utility of the combined model. The prognostic prediction of AIS was notably influenced by the baseline NIHSS score, age, as well as texture and shape features of CBF.

**Conclusion:**

The integration of clinical data and multi-PLD ASL radiomics features in a model offers a secure and dependable approach for predicting the prognosis of AIS, particularly beneficial for patients with contraindications to contrast agents. This model aids clinicians in devising individualized treatment plans, ultimately enhancing patient prognosis.

## 1 Introduction

Stroke is defined by a range of clinical syndromes featuring focal neurological impairments triggered by cerebrovascular occurrences, standing as the third leading contributor to mortality and disability worldwide (1, 2). Acute ischemic stroke (AIS) is the predominant type of stroke, accounting for approximately 87% of all cases (3). A negative prognosis confronts approximately one-third of patients with AIS, causing a marked deterioration in their quality of life and imposing a significant financial burden on society (4). The mortality and incidence rates of AIS have decreased in recent years due to advancements in medical technology. Nevertheless, the increasing global aging population could compound the existing burden (5). Therefore, it is crucial for clinicians to promptly and precisely evaluate the prognosis of AIS to tailor individualized treatment approaches.

AIS occurs when arteries become obstructed in certain regions, causing brain tissue to death due to inadequate oxygen and glucose delivery (6). The survival of brain tissue and functional recovery, as well as the prognosis of AIS, are directly influenced by cerebral blood flow (CBF) in the infarcted area (7). The evaluation of CBF is predominantly conducted through imaging modalities, such as dynamic susceptibility contrast perfusion-weighted imaging (DSC-PWI) and computed tomography perfusion (CTP) (8). However, the use of gadolinium or iodine contrast agents is limited by allergic reactions and potential renal function impairment, which restricts their widespread clinical application (9). Arterial spin labeling (ASL) is a magnetic resonance imaging (MRI) technique that quantifies CBF by applying a pulse signal to water molecules in arterial blood, serving as an endogenous contrast agent (10). Compared to traditional imaging techniques, ASL eliminates the need for exogenous contrast agents and provides several notable advantages, such as repeated usability within a short timeframe, non-radiative properties, and reduced examination costs (11). In recent years, there has been a growing utilization of ASL imaging for evaluating blood perfusion in AIS (12, 13).

Post-labeling delay (PLD) is a critical parameter in ASL technology, representing the time between the application of the labeling pulse and the acquisition of the signal (14). However, variations in arterial transit time (ATT) across brain tissues lead to a certain degree of underestimation of CBF in single-PLD ASL technology (15). To address this limitation, multi-PLD ASL technology encodes multiple PLDs within a single scan, allowing for the measurement of ATT in brain tissue and the correction of CBF for ATT (12). This effectively addresses the problem of underestimating CBF due to single-PLD scanning (16). In areas of infarction characterized by reduced blood flow, it is essential to make this correction to prevent underestimation of perfusion (17). Multi-PLD ASL has shown robust agreement with CBF measurements acquired through DSC-PWI or CTP (18–20).

Mihoko et al. employed multi-PLD ASL technology to determine the correlation between CBF and the initial severity of AIS (21). Furthermore, Li et al. discovered that the Alberta Stroke Program Early CT Score, determined through multi-PLD ASL, serves as a standalone prognostic indicator in AIS (22). Consequently, multi-PLD ASL shows promise as an alternative method for assessing the severity and prognosis of AIS. However, the quantification of CBF in the aforementioned studies offers a restricted evaluation of perfusion status and overlooks the heterogeneity present within the lesion.

Radiomics enables the rapid, objective, and high-throughput extraction of numerous features from biomedical images (23). This process reflects subtle changes that are challenging to detect visually, thereby offering a more comprehensive array of biological information (24). Guo et al. uncovered a significant correlation between the dynamic radiomics features of DSC-PWI in the infarcted region and the clinical prognosis of AIS patients at 90 days (25). However, research focusing on the ASL radiomics features of AIS patients remains limited. Therefore, we hypothesize that CBF radiomics features based on multi-PLD-ASL have significant value in the prognostic assessment of AIS patients.

In this study, our aim is to utilize machine learning (ML) techniques to construct multi-PLD ASL radiomics models for predicting the prognosis of AIS. Moreover, relevant clinical risk factors will be incorporated to improve predictive precision and determine the optimal prognostic model.

## 2 Materials and methods

### 2.1 Patients

**Figure 1:**
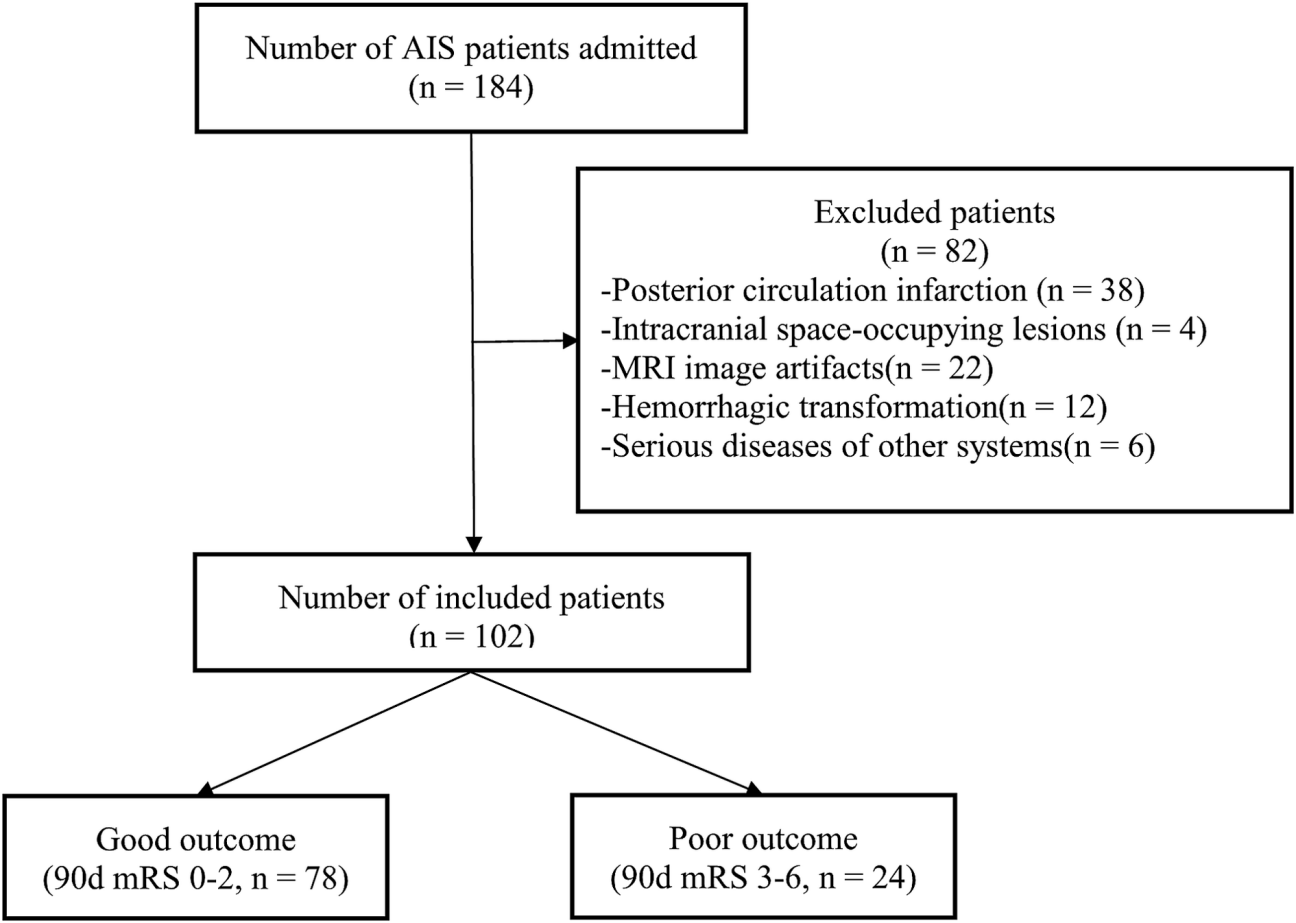
Study enrollment process. AIS, acute ischemic stroke; MRI, magnetic resonance imaging; mRS, modified Rankin Scale.

A cohort of 102 patients with AIS (45.10% female; mean [SD] age, 67.98 [12.49] years) was recruited at the Third People’s Hospital of Yancheng between December 2020 and September 2024. The screening procedures and research analysis are depicted in Figure 1.

The inclusion criteria were: (1) a confirmed diagnosis of AIS according to the guidelines (26); (2) the ability to cooperate with brain MRI scans within 24–72 h after symptom onset; (3) a modified Rankin Scale (mRS) score of 0 before symptom onset; and (4) individuals aged 18 or older.

The exclusion criteria were: (1) posterior circulation infarction; (2) intracranial occupying lesions such as tumors or arachnoid cysts; (3) significant MRI artifacts; (4) evidence of hemorrhagic transformation; and (5) patients with severe diseases in other systems.

### 2.2 Clinical assessment

Demographic and clinical data were gathered for all participants, including age, gender, onset to therapy time, baseline National Institutes of Health Stroke Scale (NIHSS) score, treatment strategies, atrial fibrillation, hypertension, diabetes mellitus, hyperlipidemia, coronary heart disease, smoking history, alcohol abuse and history of stroke. To aid in the modeling process, the continuous variables in the dataset were standardized to mitigate disparities in data distribution. The mRS score was assessed through a telephone follow-up at 90 days post-onset, classifying prognosis as either good (mRS score ≤ 2) or poor (mRS score > 2).

### 2.3 MRI acquisition

All participants underwent head MRI scans (3.0T, GE Discovery 750 W, USA), including diffusion weighted imaging (DWI) and multi-PLD ASL sequences within 24–72 h after symptom onset. Multi-PLD ASL employs a three-dimensional pseudo-continuous scanning method, with the following specific parameters: echo time: 5978 ms; repetition time: 11.5 ms; field of view: 22 × 22 cm; slice thickness: 4.5 mm; slice number: 106; resolution: 4.67 mm * 4.67 mm; NEX: 1; PLDs: 1.0 s, 1.22 s, 1.48 s, 1.78 s, 2.1 s, 2.63 s, 3.32 s; scan duration: 6 min 2 s. The ATT-corrected CBF image is obtained by averaging the individual CBF images calculated for each PLD directly on the MRI scanner.

### 2.4 Lesion segmentation and radiomics feature extraction

To improve the image quality, N4 bias field correction was applied to the obtained CBF images to minimize the impact of magnetic field inhomogeneity. Subsequently, the DWI images were used as a template to conduct image registration with the CBF images. Two radiologists independently delineated the infarct area slice-by-slice on DWI images using ITK-SNAP^1^ to define the region of interest (ROI). The ROI was then transferred to the corresponding registered CBF images. PyRadiomics software (version: 3.1.0) was utilized to extract 1,032 features from the CBF image ROI. The intraclass correlation coefficient (ICC) was computed to evaluate the consistency of the extracted features. Features with an ICC exceeding 0.75 were deemed highly consistent, standardized, and incorporated into the model development process.

### 2.5 Model building and evaluation

Eight different ML methods were employed to build models, including logistic regression, support vector machine, random forest, k-nearest neighbors, naive Bayes, extreme gradient boosting (XGBoost), light gradient boosting machine and deep neural networks. Given the limited sample size included in this study, leave-one-out cross-validation (LOOCV) was employed instead of partitioning the subjects into training and testing sets. This method involves utilizing (N-1) samples for training across N iterations, while reserving one sample as the test set in each iteration. This strategy aims to enhance data utilization and increase accuracy levels (27). In each training cycle, the least absolute shrinkage and selection operator (LASSO) regression was used to select features with non-zero coefficients through 10-fold cross-validation.

Class-weighted loss functions were utilized in model training to address the effects of class imbalance. In order to reduce bias toward the majority class (good prognosis), the minority class (poor prognosis) was assigned higher weights to amplify its influence on the loss function. Grid search was utilized for hyperparameter tuning across all models. This approach systematically investigated various combinations of model-specific hyperparameters (e.g., the penalty parameter C for logistic regression, the kernel type and gamma for support vector machine, the number of estimators and maximum depth for random forest, XGBoost, and light gradient boosting machine) to determine the optimal configuration for each method. The optimal hyperparameters were chosen according to performance metrics obtained from cross-validation.

We constructed CBF radiomics models using radiomics features and clinical models using clinical data. Furthermore, combined models were established by integrating radiomics features and clinical data. Receiver operating characteristic (ROC) curves were generated for all models to evaluate performance based on the area under the curve (AUC), sensitivity, specificity, accuracy, and F1 score. The model’s performance on the minority class (poor prognosis) was also examined through the evaluation of precision, recall, F1-score, and the area under the precision-recall AUC (PR-AUC). Additionally, the robustness of the models was validated through 5,000 permutation tests. The DeLong test was utilized to statistically compare the predictive performance of the models. Decision curve analysis (DCA) was performed to assess the potential clinical net benefits of the models. Finally, feature importance was determined within the models using SHapley Additive exPlanations (SHAP).

### 2.6 Statistical analysis

Statistical analysis was conducted using SPSS software (Version 26.0, IBM, Armonk, NY, USA). Clinical data were evaluated for normality and homogeneity of variance. Data following a normal distribution were expressed as mean ± standard deviation. In cases where the data showed a non-normal distribution, the descriptive measure employed was the median (first quartile, third quartile). Continuous variables were analyzed using the independent samples t-test or the Mann–Whitney U test, whereas categorical variables were analyzed with the chi-square test. Statistical significance was defined as *p* < 0.05.

A statistical power analysis was conducted to assess the adequacy of the sample size in supporting the study’s conclusions. The analysis was based on the observed AUC values and a null hypothesis AUC of 0.5 (random classification).

Python (version 3.11)^2^ was used for image processing and model construction. The ‘sklearn’ package was used to perform LASSO regression analysis for feature selection. The ‘matplotlib’ package was used to generate ROC and DCA curves. The ‘SHAP’ package was implemented to calculate SHAP values for the features.

## 3 Results

### 3.1 Demographic characteristics of patients

The demographic and clinical data of the included AIS patients are detailed in Table 1. Among all patients, 78 exhibited a good prognosis, while 24 had a poor prognosis. In comparison to patients with a good prognosis, those with a poor prognosis were older (*p* = 0.025) and displayed a higher baseline NIHSS score (*p* < 0.001). No significant differences were observed between the two groups in terms of gender, onset to therapy time, treatment strategies, atrial fibrillation, hypertension, diabetes mellitus, hyperlipidemia, coronary heart disease, smoking history, alcohol abuse, or history of stroke.

**Table 1:** Demographic and clinical characteristics of the good and poor prognosis groups.

Characteristics	Good (*n* = 78)	Poor (*n* = 24)	*t*/*Z*/*χ^2^*	*p* value
Age, year^a^	66 ± 12	73 ± 13	−2.283	0.025^*^
Female, n (%)	33 (42.31)	13 (54.17)	1.042	0.307
Onset to therapy time, hour^a^	13.42 ± 14.90	13.23 ± 13.89	0.055	0.956
Baseline NIHSS score^b^	3 (1, 6)	8 (4, 12)	4.505	<0.001^*^
Reperfusion therapy, n (%)	18 (23.08)	6 (25.00)	0.038	0.846
Atrial fibrillation, n (%)	12 (15.38)	5 (20.83)	0.392	0.531
Hypertension, n (%)	58 (74.36)	18 (75.00)	0.004	0.950
Diabetes mellitus, n (%)	22 (28.21)	6 (25.00)	0.095	0.758
Hyperlipidemia, n (%)	16 (20.51)	5 (20.83)	0.001	0.973
Coronary heart disease, n (%)	3 (3.85)	2 (8.33)	0.793	0.373
Smoking history, n (%)	19 (24.36)	4 (16.67)	0.622	0.430
Alcohol abuse, n (%)	13 (16.67)	3 (12.50)	0.241	0.624
History of stroke, n (%)	10 (12.82)	7 (29.17)	3.531	0.060

n, number of cases.

a (^(−x ± s).^.

b [[M (Q1, Q3)].[M (Q1, Q3)].[M (Q1, Q3)].[M (Q1, Q3)].[M (Q1, Q3)].].

NIHSS, National Institutes of Health Stroke Scale. The marked with “^*^” is statistically significant (*p* < 0.05).

### 3.2 Model building and performance

**Figure 2:**
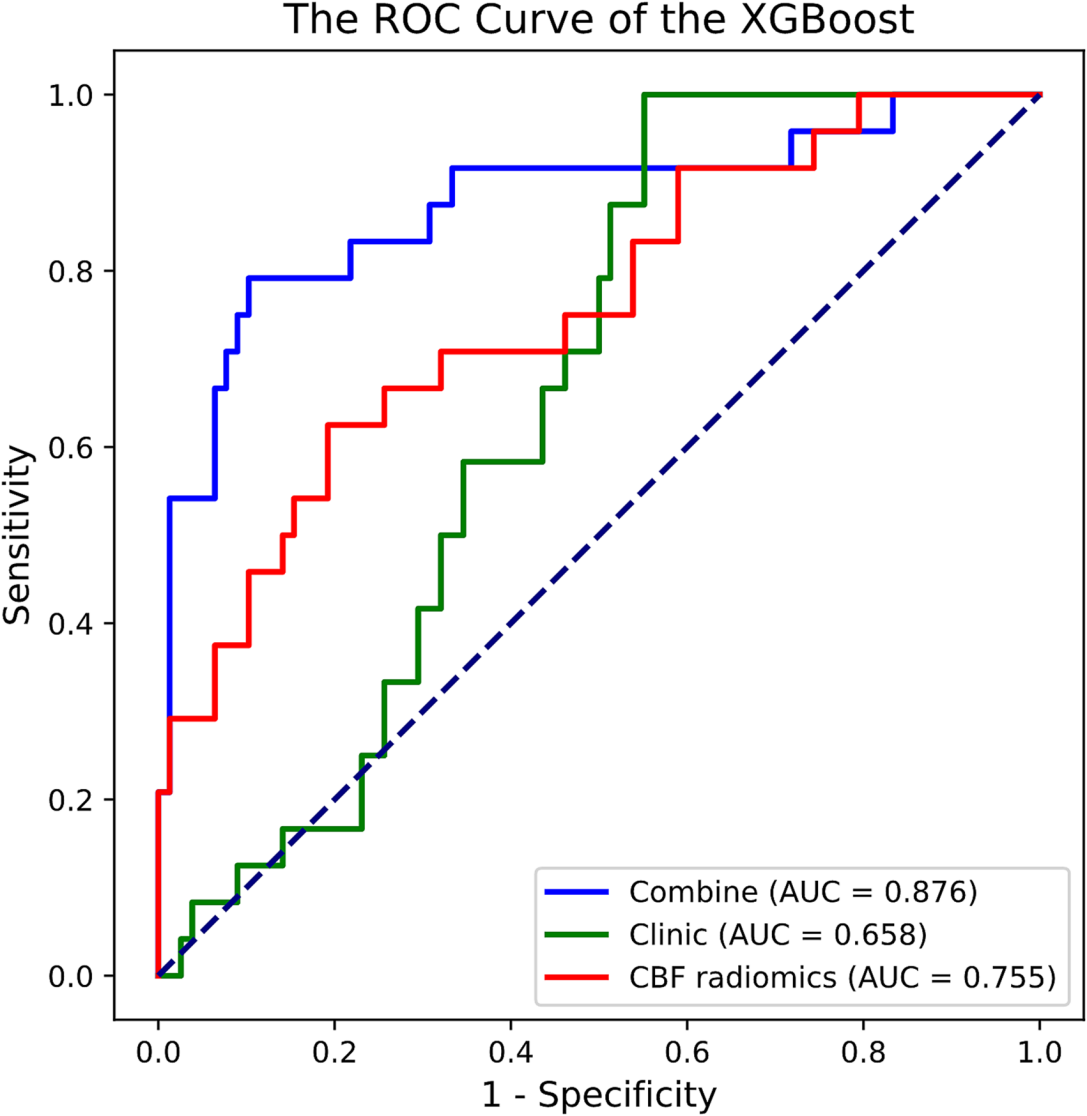
ROC curve of the XGBoost models in predicting prognosis of acute ischemic stroke. ROC, receiver operating characteristic; XGBoost, extreme gradient boosting; AUC, area under the curve; CBF, cerebral blood flow.

Out of the eight ML methods, the model constructed with the XGBoost algorithm exhibited superior performance. The hyperparameters for XGBoost were configured with a maximum depth of 6, a learning rate of 0.3, 100 estimators, a subsample of 1, and column sampling by tree set to 1. Figure 2 and Table 2 display the ROC curves and key diagnostic performance metrics for each XGBoost models. The results of the models created using the remaining seven ML algorithms are elucidated in the Supplementary material.

**Table 2:** The performance of XGBoost models in predicting prognosis of AIS patients.

Models	AUC (95% CI)	Sensitivity	Specificity	Accuracy	F1 score	Permutation test
Clinic	0.658 (0.547–0.755)	1	0.449	0.578	0.527	0.012^*^
CBF radiomics	0.755 (0.635–0.869)	0.625	0.808	0.765	0.556	0.002^*^
Combined	0.876 (0.768–0.960)	0.792	0.897	0.873	0.745	<0.001^*^

XGBoost, extreme gradient boosting; AIS, acute ischemic stroke; CBF, cerebral blood flow; AUC, area under the curve; CI, confidence interval. The marked with “^*^” is statistically significant (*p* < 0.05).

**Figure 3:**
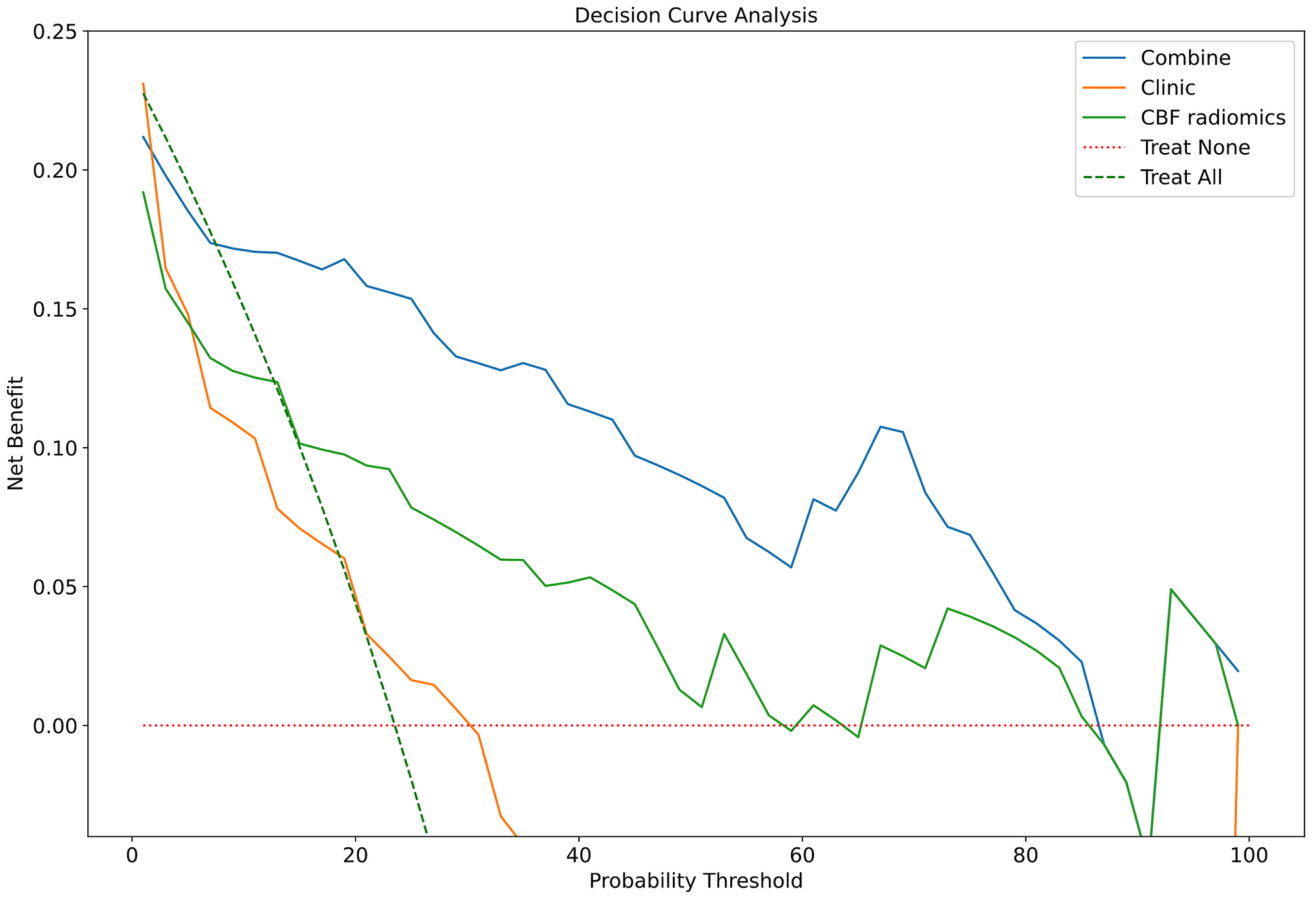
Clinical decision curve analysis of the XGBoost models. XGBoost, extreme gradient boosting; CBF, cerebral blood flow.

Among the XGBoost models, the combined model demonstrated superior performance, attaining an AUC of 0.876 (95% CI, 0.768–0.960), indicating its significant capability in discriminating between good and poor prognoses. In comparison, the AUC for the clinical model was 0.658 (95% CI, 0.547–0.755), while the AUC of the CBF radiomics model was 0.755 (95% CI, 0.635–0.869). The DeLong test demonstrated that the combined model exhibited markedly better predictive performance compared to both the clinical model (*p* < 0.001) and the CBF radiomics model (*p* = 0.002). Furthermore, there was no statistically significant difference between the clinical model and the CBF radiomics model (*p* = 0.222). During the 5,000 permutation tests, it is noteworthy that all models exhibited a relatively high level of robustness. In Figure 3, the DCA for all models reveals that the combined model exhibits superior net benefit compared to both the clinical model and the CBF radiomics model across a broad spectrum of threshold probabilities, ranging from 0.1 to 0.9.

Based on the observed AUC of the combined model (AUC = 0.876) and a null hypothesis AUC of 0.5, a statistical power analysis resulted in a standard error of 0.034 and a *Z*-value of 10.970 (*p* < 0.05), demonstrating that the current dataset possesses adequate power (>80%) to validate the observed AUC. Furthermore, the combined model achieved a precision of 0.79, recall of 0.83, PR-AUC of 0.85, and F1-score of 0.81 for the minority group, indicating its ability to accurately identify cases with poor prognosis.

### 3.3 Model interpretability

**Figure 4:**
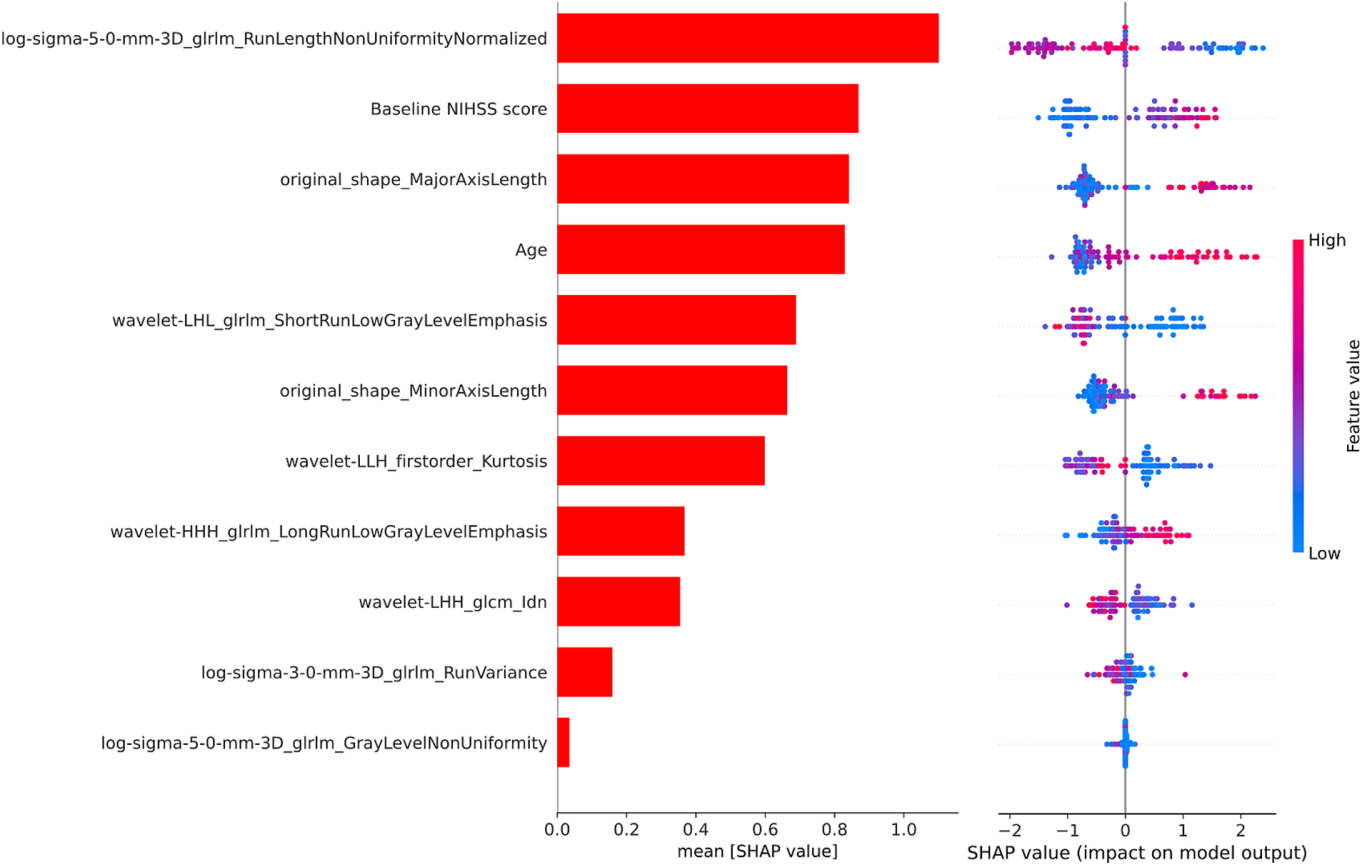
The SHAP values of the XGBoost combined model. SHAP, SHapley Additive Explanations; XGBoost, extreme gradient boosting; NIHSS, National Institutes of Health Stroke Scale.

As illustrated in Figure 4, we used the SHAP values from the best-performing XGBoost combined model to identify the important variables for predicting AIS prognosis. Positive SHAP values were linked to an elevated risk of poor prognosis, while negative SHAP values were indicative of a greater chance of poor prognosis. Notably, the baseline NIHSS score and age played significant roles in predicting the prognosis of AIS. Additionally, several texture and shape features derived from the CBF images also contributed significantly to the predictive performance of the model.

## 4 Discussion

This study proposes a novel approach that integrates multi-PLD ASL radiomics features with clinical variables for predicting AIS prognosis. Among the models developed, the XGBoost algorithm exhibited the highest AUC value of 0.876, underscoring its predictive accuracy for AIS prognosis. It can assist clinicians in early prognostic assessment and the implementation of personalized treatment plans, ultimately aiming to reduce the incidence of poor prognosis.

The fundamental aspect of AIS involves tissue necrosis caused by embolism in the supplying artery. CBF serves as a direct reflection of the hemodynamic condition within the infarcted region, making it a pivotal indicator of brain tissue injury and a key role in evaluating neurological rehabilitation (28). In contrast to conventional DSC-PWI, multi-PLD ASL technology produces CBF images without the need for contrast agents (29). Moreover, radiomics techniques enable the extraction of multidimensional features from medical images, providing a more thorough assessment of the heterogeneity of blood perfusion within lesions in comparison to visual evaluation or basic CBF quantification (30). Guo et al. developed ten ML models based on the dynamic radiomics features of DSC-PWI in the infarcted area to predict the prognosis of AIS, achieving a maximum AUC of 0.882 (25). However, there is a scarcity of studies investigating the correlation between the radiomics features of CBF in the infarcted region and the prognosis of AIS. Therefore, we aimed to develop a CBF radiomics model for AIS prognosis prediction based on multi-PLD ASL imaging. Our model attained a maximum AUC of 0.755 in the results, indicating the viability of utilizing multi-PLD ASL technology for predicting neurological recovery in AIS patients. After including age and baseline NIHSS score, the model achieves an AUC of 0.876, which aligns closely with the findings of Guo et al.’s model (25). Therefore, this method presents a safer and more reproducible avenue for prognostic prediction in AIS patients, particularly for those with contraindications to contrast agents. Moreover, the absence of contrast agents renders ASL a more economical method.

When examining the demographic and clinical profiles of AIS patients, it was observed that those with poorer prognoses were characterized by advanced age and a higher baseline NIHSS score. The development of a predictive model utilizing demographic and clinical factors yielded limited performance, as indicated by an AUC of 0.658, which falls below that of the CBF radiomics model. While the DeLong test did not indicate a statistically significant distinction between the two models, the test’s power may have been constrained by the small sample size (31). Consequently, there is justification to argue for the superiority of the CBF radiomics model compared to the clinical model. During the acute phase of AIS, the CBF radiomics model may function as an alternative to the clinical model for prognostic prediction, especially beneficial for patients with insufficient clinical data.

The combined model, which integrates clinical data with CBF radiomics features, achieved an AUC of 0.876. The DeLong test demonstrated that the combined model significantly outperformed both the clinical model and the CBF radiomics model. This finding implies that clinical data provide only partial insight into prognosis. In contrast, CBF radiomics features provide distinct information on alterations in cerebral blood perfusion status that clinical data alone cannot encompass. This discovery is in line with results from various studies, underscoring the essential role of the complementarity of multimodal information in radiomics research (32). Additionally, the DCA demonstrated that the combined model yielded a higher net benefit across a wide range of threshold probabilities. This suggests that the model has the potential for application in clinical practice, with the only requirement being the extraction of relevant clinical and imaging data from electronic medical records and imaging systems. It can assist clinicians in delivering consistent prognostic evaluations in diverse clinical scenarios, enhancing the management of AIS by facilitating prompt interventions and optimizing resource distribution.

In order to determine the factors influencing the prognosis of AIS, we conducted SHAP analysis to assess the individual contribution of each feature to the predictive accuracy of the combined model. The baseline NIHSS score and age emerged as the most significant predictors among the clinical variables, aligning with prior studies (33–35). The baseline NIHSS score reflects stroke severity, correlating strongly with infarct size and functional outcomes in AIS patients (36). Advanced age, characterized by diminished neural plasticity and a higher prevalence of comorbidities, is a firmly established risk factor for poor outcomes in AIS (37, 38). Texture features and shape features derived from multi-PLD ASL CBF were identified as important contributors to the model. Among the texture features, the gray level run length matrix made the greatest contribution, quantifying gray intensity patterns and their spatial relationships within the ischemic area (39). Ischemic regions often exhibit varying degrees of blood flow reduction and tissue damage, and texture features can capture these variations, distinguishing areas of hypoperfusion, penumbra, and infarct core. Moreover, alterations in tissue density induced by cytotoxic and vasogenic edema may manifest in texture characteristics, elucidating the advancement of secondary damage (40). Additionally, microstructural disruptions, such as the loss of neural integrity or capillary breakdown, may manifest as increased texture irregularities in radiomics analysis (41). Shape features provide evidence linking lesion geometry to the underlying vascular anatomy and collateral perfusion (42). Larger or irregularly shaped lesions may indicate more extensive vascular occlusion or failure of collateral circulation, both of which are associated with poor prognosis (43, 44). Moreover, regions with poor perfusion are more prone to displaying irregular lesion morphologies (45). These findings highlight the capability of CBF radiomics features to reflect the extent and heterogeneity of tissue damage, demonstrating their relevance to clinical prognosis of AIS.

This study must acknowledge several limitations. Firstly, the small sample size may restrict the generalizability of the results. Despite utilizing LOOCV and permutation tests to mitigate this issue, larger multi-center datasets are essential to confirm the model and broaden its external applicability. Secondly, the exclusion of posterior circulation infarction cases restricted the model’s generalizability. Future research should incorporate posterior circulation cases into larger datasets to construct subtype-specific models. Thirdly, despite efforts to ensure consistency through ICC analysis, manual delineation of infarcted areas may lead to observer variability (46). Future research should explore automated or semi-automated segmentation methods to reduce subjectivity and improve reproducibility. Fourthly, this study focused exclusively on the multi-PLD ASL sequence. Future studies should explore integrating multimodal imaging techniques, such as quantitative susceptibility mapping and diffusion-prepared ASL, to better understand AIS progression by delineating the ischemic penumbra and assessing blood–brain barrier integrity (47–50). Finally, future studies could integrate radiomics features with network-based approaches, which have proven valuable in neuropsychiatric disorders, to better understand how ischemic lesions disrupt brain networks and influence prognosis of AIS (51–53).

## 5 Conclusion

In this study, we established new prognostic prediction models for AIS utilizing multi-PLD ASL technology. The results indicate that the combined model, which integrates clinical data and CBF radiomics features, accurately predicts AIS prognosis. This model has the potential to assist clinicians in identifying individualized treatment approaches to improve patient prognosis. This strategy presents a feasible alternative for assessing the prognosis of AIS, especially in individuals for whom the use of contrast agents is contraindicated.

## Data Availability

The original contributions presented in the study are included in the article/Supplementary material, further inquiries can be directed to the corresponding authors.

## Footnotes

^1^http://www.itk-snap.org, version 4.0.

^2^ https://www.python.org
