# Supplementary material for: Prognostic value of multi-PLD ASL radiomics in acute ischemic stroke
Source: Front Neurol. 2025 Jan 13;15:1544578. doi: 10.3389/fneur.2024.1544578 (PMC11769822; doi:10.3389/fneur.2024.1544578)
Supplement: Supplementary file 1 [file Table_1.DOCX]

**Supplementary material**

**Supplementary figure. ROC curves of eight machine learning models for predicting AIS prognosis.**

**
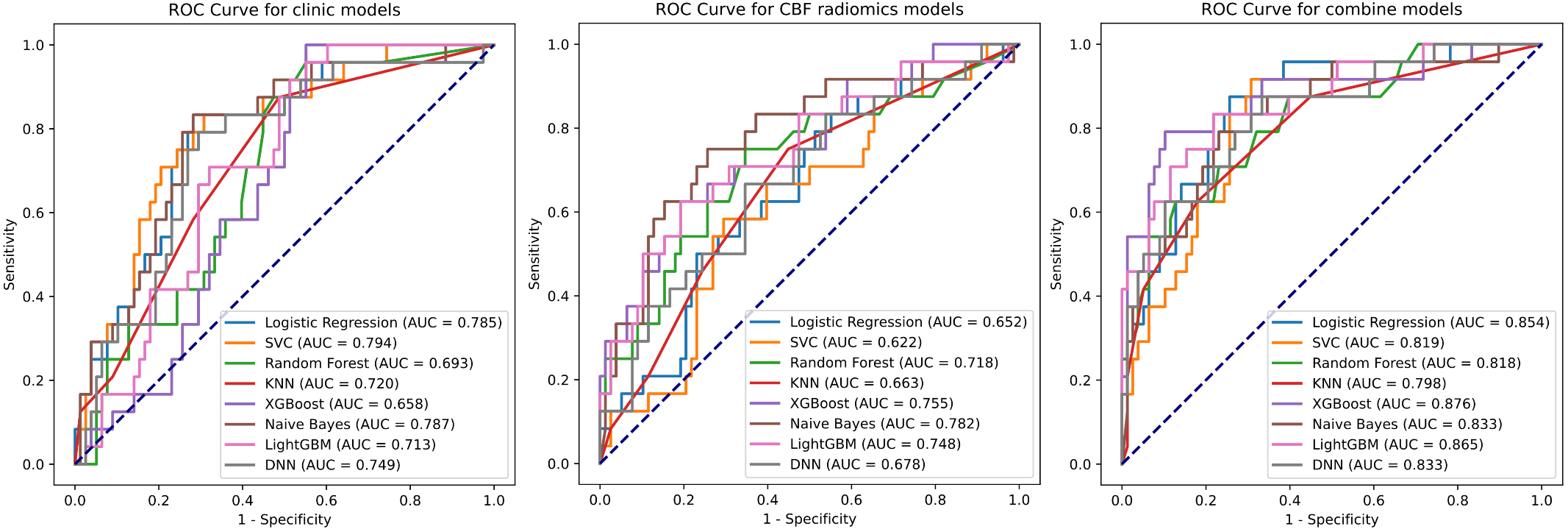
**

ROC, receiver operating characteristic; AIS, acute ischemic stroke; CBF, cerebral blood flow; AUC, area under the curve; SVC, support vector machine; KNN, k-nearest neighbors; XGBoost, extreme gradient boosting; LightGBM, light gradient boosting machine; DNN, deep neural networks.

**Supplementary table. The performance of eight machine learning models in predicting prognosis of AIS patients.**

| Models | | AUC (95% CI) | Sensitivity | Specificity | Accuracy | F1 score |
| --- | --- | --- | --- | --- | --- | --- |
| Clinic | Logistic Regression | 0.785(0.683-0.870) | 0.833 | 0.692 | 0.725 | 0.588 |
|  | SVC | 0.794(0.692-0.879) | 0.833 | 0.692 | 0.725 | 0.588 |
|  | Random Forest | 0.693(0.578-0.792) | 0.958 | 0.449 | 0.569 | 0.511 |
|  | KNN | 0.720(0.606-0.821) | 0.875 | 0.513 | 0.598 | 0.506 |
|  | XGBoost | 0.658(0.547-0.755) | 1 | 0.449 | 0.578 | 0.527 |
|  | Naive Bayes | 0.787(0.682-0.874) | 0.833 | 0.718 | 0.745 | 0.606 |
|  | LightGBM | 0.713(0.611-0.808) | 0.958 | 0.449 | 0.569 | 0.511 |
|  | DNN | 0.749(0.631-0.844) | 0.792 | 0.705 | 0.725 | 0.576 |
| CBF radiomics | Logistic Regression | 0.652(0.527-0.782) | 0.833 | 0.449 | 0.539 | 0.460 |
|  | SVC | 0.622(0.490-0.761) | 0.583 | 0.705 | 0.676 | 0.459 |
|  | Random Forest | 0.718(0.589-0.846) | 0.750 | 0.654 | 0.676 | 0.522 |
|  | KNN | 0.663(0.548-0.789) | 0.750 | 0.551 | 0.598 | 0.468 |
|  | XGBoost | 0.755(0.635-0.869) | 0.625 | 0.808 | 0.765 | 0.556 |
|  | Naive Bayes | 0.782(0.661-0.889) | 0.750 | 0.744 | 0.745 | 0.581 |
|  | LightGBM | 0.748(0.631-0.866) | 0.625 | 0.808 | 0.765 | 0.556 |
|  | DNN | 0.678(0.553-0.806) | 0.667 | 0.654 | 0.657 | 0.478 |
| Combine | Logistic Regression | 0.854(0.762-0.934) | 0.875 | 0.744 | 0.775 | 0.646 |
|  | SVC | 0.819(0.714-0.907) | 0.917 | 0.692 | 0.745 | 0.629 |
|  | Random Forest | 0.818(0.711-0.912) | 0.625 | 0.872 | 0.814 | 0.612 |
|  | KNN | 0.798(0.692-0.901) | 0.625 | 0.821 | 0.775 | 0.566 |
|  | XGBoost | 0.876(0.768-0.960) | 0.792 | 0.897 | 0.873 | 0.745 |
|  | Naive Bayes | 0.833(0.730-0.923) | 0.792 | 0.769 | 0.775 | 0.623 |
|  | LightGBM | 0.865(0.768-0.944) | 0.833 | 0.782 | 0.794 | 0.656 |
|  | DNN | 0.833(0.729-0.919) | 0.875 | 0.667 | 0.716 | 0.592 |

AIS, acute ischemic stroke; CBF, cerebral blood flow; AUC, area under the curve; CI, confidence interval; SVC, support vector machine; KNN, k-nearest neighbors; XGBoost, extreme gradient boosting; LightGBM, light gradient boosting machine; DNN, deep neural network.
